# Direct Healthcare Cost of Obesity in Brazil: An Application of the Cost-of-Illness Method from the Perspective of the Public Health System in 2011

**DOI:** 10.1371/journal.pone.0121160

**Published:** 2015-04-01

**Authors:** Michele Lessa de Oliveira, Leonor Maria Pacheco Santos, Everton Nunes da Silva

**Affiliations:** University of Brasilia, Brasília, Federal District, Brazil; London School of Hygiene and Tropical Medicine, UNITED KINGDOM

## Abstract

**Background:**

Obesity is a global public health problem and a risk factor for several diseases that financially impact healthcare systems.

**Objective:**

To estimate the direct costs attributable to obesity (body mass index {BMI} ≥ 30 kg/m^2^) and morbid obesity (BMI ≥ 40 kg/m^2^) in adults aged ≥ 20 incurred by the Brazilian public health system in 2011.

**Settings:**

Public hospitals and outpatient care.

**Methods:**

A cost-of-illness method was adopted using a top-down approach based on prevalence. The proportion of the cost of each obesity-associated comorbidity was calculated and obesity prevalence was used to calculate attributable risk. Direct healthcare cost data (inpatient care, bariatric surgery, outpatient care, medications and diagnostic procedures) were extracted from the Ministry of Health information systems, available on the web.

**Results:**

Direct costs attributable to obesity totaled US$ 269.6 million (1.86% of all expenditures on medium- and high-complexity health care). The cost of morbid obesity accounted for 23.8% (US$ 64.2 million) of all obesity-related costs despite being 18 times less prevalent than obesity. Bariatric surgery costs in Brazil totaled US$ 17.4 million in 2011. The cost of morbid obesity in women was five times higher than it was in men.

**Conclusion:**

The cost of morbid obesity was found to be proportionally higher than the cost of obesity. If the current epidemic were not reversed, the prevalence of obesity in Brazil will increase gradually in the coming years, as well as its costs, having serious implications for the financial sustainability of the Brazilian public health system.

## Introduction

The obesity epidemic is recognized by the World Health Organization as a public health problem. Healthcare expenditure on this chronic disease is known to rise in direct proportion to body mass index (BMI) [1]. In 2008, 502 million adults (aged 20+) were obese worldwide [2], with a 10% prevalence in men and 14% in women. The prevalence of obesity is even higher in the Americas – 26% in both sexes – and is on the rise, having doubled between 1980 and 2008 [3]. According to the Brazilian Consumer Expenditure Survey conducted in 2008–2009, obesity affects 14.8% of the Brazilian population, including 12.5% of adult men and 16.9% of adult women. Obese individuals represent nearly 25% of all overweight men and nearly a third of all overweight women [4]. The prevalence of overweight and obesity has increased over the last four decades. Morbid obesity (body mass index {BMI} ≥ 40 kg/m^2^) is rising at a higher rate than obesity in Brazil. Between 1989 and 2003, its prevalence in men quadrupled (from 0.08% to 0.32%), while its prevalence in women increased by 44% (from 0.66% to 0.95%), but it still remained higher in women [5].

2.8 million people die each year around the world as a result of being overweight or obese [6]. Obese people have a higher risk of type 2 diabetes and cardiovascular diseases such as coronary heart disease and strokes, as well as hypertension, osteoarthritis and several types of cancer, including colorectal, breast, uterine and renal cancer [7–14]. When the relative risk of a disease is higher for obese people, a portion of cases can be directly attributed to obesity [15]. In the BMI range of 30–35 kg/m², average survival is reduced by 2 to 4 years; in the 40–45 kg/m² range, it is reduced by 8 to 10 years [16], or by 5 to 20 years depending on sex, age and ethno-racial group [17]. A study conducted in the United States estimated that 14% of all cancer deaths and 20% of women’s deaths are related to obesity [18]. The higher the BMI, the greater is the risk of comorbidity [19–22].

Overweight and obesity contribute to the global burden of disease [23]. In 2002, the proportion of the disease burden attributable to a higher BMI was 58% for type 2 diabetes, 21% for ischemic heart disease, 39% for hypertension, 23% for stroke, 12% for colon cancer, 8% for postmenopausal breast cancer, 32% for endometrial cancer and 13% for osteoarthritis [24]. In this regard, there is undeniably a connection between rising rates of obesity and rising medical spending [25], as well as disability-adjusted life years (DALYs) and years lost due to disability (YLDs).

Studies that emphasize the disproportionality of the cost of treating people with different degrees of obesity can assist health managers in making decisions about how to allocate resources [26, 27] dedicated to preventing and treating this disease. Accordingly, this study aimed to estimate the direct costs attributable to obesity (BMI ≥ 30 kg/m^2^) and morbid obesity (BMI ≥ 40 kg/m^2^) in adults aged 20 and over incurred by the Brazilian public health system in 2011.

## Materials and Methods

A prevalence-based cost-of-illness analysis was adopted with a top-down approach. We carried out the analysis from the perspective of public healthcare services, estimating direct healthcare costs attributable to obesity and morbid obesity in Brazil.

A search was conducted of all obesity-related outpatient and inpatient costs. These amounts were added to the costs of other diseases that are related to obesity – diseases and conditions that could have been avoided if obesity had been prevented [28, 29]. These costs were obtained by calculating the proportion of total disease cases associated with obesity (Population Attributable Risk – PAR) and multiplying this measure by the cost of treating these comorbidities. The PAR for obesity was calculated for each disease and categorized by BMI range and sex. PAR was calculated using the following formula [30]:

$$PAR=\frac{P\;(RR-1)}{P\left(RR-1\right)+1}\;$$

in which:

- P = prevalence of obese (or morbidly obese) individuals, obtained from the 2008–2009 Consumer Expenditure Survey [4], and
- RR = relative risk for a certain disease in obese (or morbidly obese) individuals vs. eutrophic individuals.

To calculate the PAR, relative risks (RR) were used (and their confidence intervals) obtained when the risk of incidence of each disease in obese (or morbidly obese) patients was compared to the risk of incidence in normal weight patients. The latter data were obtained in cohort studies and meta-analyses published in international journals after 2000 and indexed in the following databases: *Medline*, *Evidence-Based Medicine Reviews (EBMR)*, *Cochrane Database of Systematic Reviews*, Lilacs, Scielo and Medilene via Pubmed. When the RR was not found, the risk was estimated using the odds ratio (OR). If the outcome is relatively rare in population (10% or less), the relative risk could be estimated by odds ratio [31, 32]. The meta-analyses adopted for the RR or OR were identified by Pubmed and selected with the AMSTAR [33] tool, which enables the assessment of systematic reviews. Most of the studies found were conducted in the United States and Europe. One of the main sources of relative risk for this study was Guh et al 2009 [9], who used the following criteria to select studies for inclusion in their meta-analysis: prospective cohort study of the population of a Western country, relevant results, sample size of at least 200 individuals and risk estimate based on disease incidence rather than disease mortality rate.

Obesity prevalence was obtained from the Consumer Expenditure Survey (Pesquisa de Orçamentos Familiares in Portuguese), which employed a nationally representative sample of the Brazilian households carried out by the Brazilian Institute of Geography and Statistics (IBGE, acronym in Portuguese). Data from 55,970 households and 188,461 respondents were collected between 2008 and 2009. Weight and height were measured using a portable electronic scale and a portable stadiometer, respectively [4].

Costs related to hospital (inpatient) and ambulatory (outpatient) care were included in this study. The following direct healthcare costs were obtained: medical consultations, inpatient care, bariatric surgeries, etc.; the cost of medium- and high-complexity procedures, including complementary procedures (clinical pathology, radiology, ultrasound exams, nuclear medicine, interventional radiology and computerized tomography); and the cost of medications, orthotics and prosthetics provided by the Brazilian public health system. This information, which was organized according to the chapters of the International Disease Classification (ICD-10 – WHO, 1995), was obtained from the Brazilian Health Ministry’s Hospital Information System and Outpatient Information System. The identification of the procedures related to obesity, morbid obesity and associated diseases was made through the ICD-10, being registered in the Table Management System of Procedures, Medical drugs, Orthotics, Prosthetics and Special Materials of the Unified Health System (SIGTAP in Portuguese), available in the Health Information Systems of the Ministry of Health. These databases correspond to all procedures performed in the public health system and financially supported by the Ministry of Health. Therefore, they included all people living in Brazil that accessed the public health system, all age groups and both sexes. The public system is responsible for approximately 75% of medical care in Brazil [34].

As this study is an analysis of the public health service’s costs, we did not include family expenses such as transportation, food (including dietary products), lodging or patient care by family providers.

The study protocol was reviewed and approved by the Ethics in Research Committee of the Faculty of Health Sciences of University of Brasilia (report no. 120-10). All data came from secondary database without identifying people.

## Results

### Comorbidities associated with obesity

**Fig 1:**
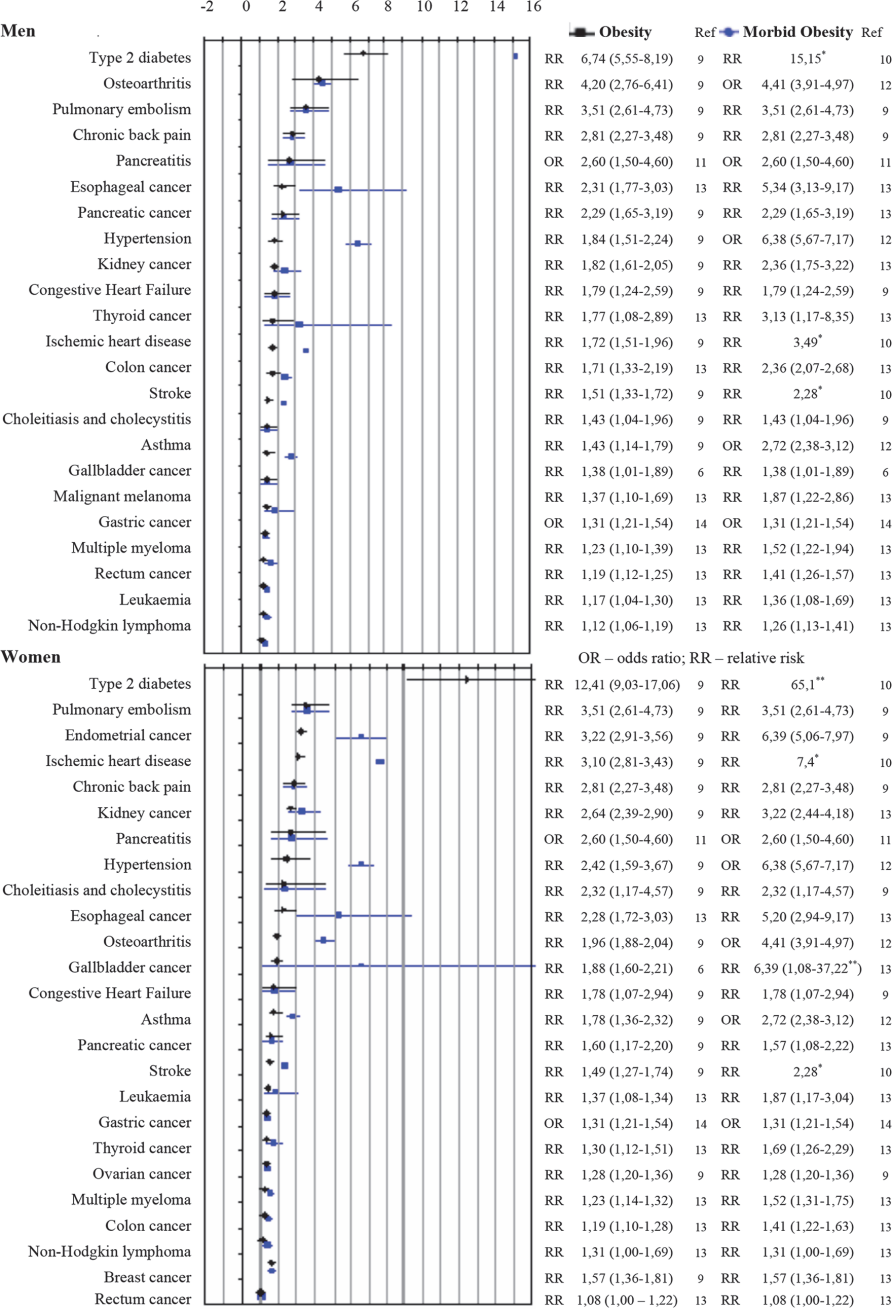
RR or OR for diseases associated with obesity (BMI ≥ 30 kg/m^2^) and morbid obesity (BMI ≥ 40 kg/m^2^) in adults aged 20 and over, according to sex. Sources: ^6^WHO 2012, ^9^Guh, 2009, ^10^IASO (1) (*), ^11^Martinez, 2004, ^12^Mokdad et al, 2001, ^13^Renehan et al, 2008 (1), ^14^Yang, 2009. (1) RR calculated for the mean BMI of morbidly obese adults (BMI = 43.42 kg/m^2^) (*) In this study confidence interval for the RR were not calculated (**) outliers

Fig 1 shows the diseases analyzed in this study, as well as their relative risk or odds ratio related to obesity and morbid obesity. Diabetes is a disease with a higher risk of incidence in people with obesity or morbid obesity. Besides diabetes, the diseases with the greatest risk of incidence in obese men are osteoarthritis and pulmonary embolism, while the greatest risk of incidence in obese women is for pulmonary embolism and endometrial cancer. The pathologies with increased risk of incidence in men with morbid obesity (besides diabetes) are hypertension and esophageal cancer; in women, they are endometrial cancer, gallbladder cancer and hypertension (Fig 1). An association between obesity and skin cancer was only found in men [13], and an association was also found between obesity and three female cancers: ovarian, breast and endometrial. Table 1 reports the population attributable risks (PAR) calculated for some diseases associated with obesity and morbid obesity, according to sex.

**Table 1:** Population attributable risk (PAR) for diseases associated with obesity and morbid obesity in adults aged 20 and over, according to sex. Brazil, 2011.

Diseases associated		PAR – Obesity (BMI ≥ 30 kg/m^2^)						PAR – Morbid obesity (BMI ≥ 40 kg/m^2^)					
	ICD-10	Men	Men		Women	Women		Men	Men		Women	Women	
			95% CI			95% CI			95% CI			95% CI	
Asthma	J45	0.051	0.0172	0.0899	0.1165	0.0574	0.1824	0.0075	0.006	0.0092	0.0192	0.0155	0.0236
Breast cancer	C50, D05, D24, D48.6	-	-	-	0.0215	0.0084	0.0358	-	-	-	0.0065	0.0041	0.0091
Cholelithiasis and cholecystitis	K80, K81	0.051	0.005	0.1071	0.1824	0.0279	0.3763	0.0019	0.0002	0.0042	0.0148	0.0019	0.0391
Chronic back pain	M54	0.2599	0.221	0.3031	0.322	0.2773	0.3703	0.0079	0.0056	0.0108	0.0202	0.0143	0.0275
Colon cancer	C18, D12.0—D12.6	0.0815	0.0396	0.1295	0.0308	0.017	0.0447	0.0059	0.0047	0.0073	0.0047	0.0025	0.0071
Congestive Heart Failure	I50.0	0.0899	0.0291	0.1658	0.1165	0.0117	0.2469	0.0035	0.0011	0.0069	0.0088	0.0008	0.0216
Endometrial cancer	C54.1, C55, D07.0, D39.0	-	-	-	0.2728	0.244	0.302	-	-	-	0.0579	0.0442	0.0736
Esophageal cancer	C15, D00.1	0.1407	0.0877	0.202	0.178	0.108	0.255	0.0187	0.0093	0.0347	0.0457	0.0216	0.0852
Gallbladder cancer	C23, C24, D13.5	0.0453	0.0012	0.1001	0.1295	0.0921	0.1698	0.0017	0	0.0039	0.0579	0.0009	0.2922
Gastric cancer	C16, D00.2, D13.1	0.0373 (0.0256–0.0632)						0.0014	0.0009	0.0024	0.0035	0.0024	0.0061
Hypertension	I10—I15	0.095	0.0599	0.1342	0.1935	0.0907	0.3109	0.0231	0.0201	0.0264	0.0578	0.0505	0.0657
Ischemic heart disease^a^	I20—I25	0.0826	0.0599	0.1071	0.2619	0.2342	0.2911	0.0108	-	-	0.068	-	-
Kidney cancer	C64-C66, D30.0-D30.2	0.093	0.0708	0.116	0.217	0.1902	0.2431	0.0059	0.0033	0.0097	0.0247	0.0162	0.035
Leukemia	C91—C95	0.0208	0.005	0.0361	0.0589	0.0133	0.0543	0.0016	0.0004	0.003	0.0098	0.0019	0.0227
Malignant melanoma	C43, D03	0.0442	1.0442	2.0442	-	-	-	0.0038	0.001	0.0081	-	-	-
Multiple myeloma	C90	0.0279	0.0123	0.0465	0.0374	0.0231	0.0513	0.0023	0.001	0.0041	0.0059	0.0035	0.0085
Non-Hodgkin lymphoma	C82, C83, C85	0.0148	0.0074	0.0232	0.0231	0	0.0483	0.0011	0.0006	0.0018	0.0035	0	0.0078
Osteoarthritis	M15—M19	0.2857	0.1803	0.4034	0.1396	0.1295	0.1495	0.0148	0.0126	0.0172	0.0374	0.0321	0.0433
Ovarian cancer	C56, D27, D39.1	-	-	-	0.0452	0.0327	0.0574	-	-	-	0.0032	0.0023	0.0041
Pancreatic cancer	C25, D01.7, D13.6, D13.7	0.1389	0.0751	0.2149	0.0921	0.0279	0.1686	0.0056	0.0029	0.0095	0.0068	0.0019	0.0135
Pancreatitis	K85, K86	0.1915 (0.0689–0.3476)						0.007	0.0022	0.0156	0.0179	0.0057	0.0394
Pulmonary embolism	I26	0.2388	0.1675	0.318	0.2978	0.2139	0.3866	0.0109	0.007	0.0161	0.0278	0.018	0.0408
Rectum cancer	C19, C20, D12.7-D12.9	0.0232	0.0148	0.0303	0.0068	0	0.017	0.0018	0.0011	0.0025	0.0009	0	0.0025
Stroke^a^	I64, 169.4	0.0599	0.0396	0.0826	0.0765	0.0436	0.1112	0.0056	-	-	0.0144	-	-
Thyroid cancer	C73, D34, D44.0	0.0878	0.0099	0.1911	0.0483	0.0199	0.0794	0.0093	0.0007	0.0313	0.0078	0.003	0.0145
Type 2 diabetes^a^	E11, E13, E14	0.4178	0.3625	0.4733	0.6585	0.5757	0.7308	0.0586	-	-	0.4222	-	-

Sources: ^6^WHO 2012, ^9^Guh, 2009, ^10^IASO, ^11^Martinez, 2004, ^12^Mokdad et al, 2001, ^13^Renehan et al, 2008, ^14^Yang, 2009. Prevalence of obesity and morbid obesity were calculated from Ref. 4.Brasil Consumer Expenditure Survey 2008–2009.

^a^ In this study the CI 95% for the RR relative risk obtained for morbid obesity was not calculated

### Costs associated with obesity

Table 2 presents the main results of this study: the costs attributable to obesity and morbid obesity, which incurred on the Brazilian public health system budget in 2011, according to associated diseases. These costs totaled US$ 269.6 million, which corresponded to 1.86% of all Ministry of Health’s expenditure related to hospital and ambulatory care in Brazil. Comparing the costs by healthcare level, 59.2% of the total cost was hospital admissions (inpatient care) and 40.8% corresponded to ambulatory procedures (outpatient care). When analyzed by associated disease, obesity-attributable costs were highest for ischemic heart disease, followed by breast cancer, congestive heart failure and diabetes (Table 2).

**Table 2:** Total costs attributable to obesity and morbid obesity in the adult population aged 20 and over, according to associated diseases, Brazil, 2011.

Diseases associated	Costs attributable to obesity (BMI ≥ 30 kg/m^2^) (in thousand US$)			Costs attributable to morbid obesity (BMI ≥ 40 kg/m^2^) (in thousand US$)			Proportion attributable to morbid obesity in relation to the total costs of obesity (%)
	Total	95% CI		Total	95% CI		
Asthma	3,750	1,676	6,090	599	482	735	16.0
Breast cancer	16,934	6,616	28,197	5,085	3,220	7,207	30.0
Cholelithiasis and cholecystitis	12,734	1,894	26,306	992	128	2,602	7.8
Chronic back pain	10,835	9,292	12,519	565	399	769	5.2
Colon cancer	14,103	7,124	21,845	1,347	908	1,846	9.6
Congestive Heart Failure	16,280	3,275	32,482	959	148	2,228	5.9
Endometrial cancer	9,000	8,050	9,964	1,909	1,459	2,428	21.2
Esophageal cancer	7,348	4,545	10,553	1,228	597	2,281	16.7
Gallbladder cancer	602	356	884	225	3	1,130	37.5
Gastric cancer	2,296	1,576	3,891	130	89	227	5.7
Hypertension	3,851	1,976	5,982	1,091	954	1,243	28.3
Ischemic heart disease	91,806	77,015	107,603	19,977	-	-	21.8
Kidney cancer	2,650	2,220	3,083	257	162	376	9.7
Leukemia	13,253	3,052	15,547	1,839	370	4,131	13.9
Malignant melanoma	508	12,004	23,501	44	11	93	8.6
Multiple myeloma	1,793	971	2,689	224	122	345	12.5
Non-Hodgkin lymphoma	2,813	4,636	8,421	334	49	678	11.9
Obesity (bariatric surgery)	17,710	-	-	17,394	-	-	98,2
Osteoarthritis	7,376	5,687	9,225	1,134	973	1,313	15.4
Ovarian cancer	2,878	2,082	3,655	202	145	260	7.0
Pancreatic cancer	4,188	1,858	6,963	227	87	420	5.4
Pancreatitis	2,252	810	4,089	134	42	294	5.9
Pulmonary embolism	1,280	912	1,676	99	64	145	7.7
Rectum cancer	2,013	1,023	3,122	181	79	320	9.0
Stroke	5,677	3,476	8,055	819	-	-	14.4
Thyroid cancer	712	228	1,287	103	32	225	14.5
Type 2 diabetes	14,959	13,045	16,718	7,073	-	-	47.3
Total	269,601	175,399	374,345	64,172	-	-	23.8

(1US$ = R$1.81)

### Costs according to severity of obesity

This study was the first one to estimate morbid obesity costs in Brazil, which corresponded to $64.2 million in 2011. The highest costs attributable to morbid obesity were for ischemic heart disease and diabetes, probably because ischemic heart disease treatment is expensive and diabetes is a disease that has the highest incidence risk in people with obesity or morbid obesity. Despite the fact that morbid obesity was 18 times less prevalent than obesity, morbid obesity costs accounted for 23.8% of all obesity-related costs. Bariatric surgery was the single highest cost, accounting for $17.4 million, or 27.1% of this total. Between 2008 and 2011, the number of bariatric surgeries increased annually, as did the annual cost of such procedures (Table 3).

**Table 3:** Evolution of the number and annual cost (US$) of procedures related to bariatric surgery in the public health system, Brazil, 2008–2011.

Year	Number of bariatric surgeries	Annual cost (US$)
2008	3,139	9,445,275.83
2009	3,681	12,338,585.09
2010	4,441	14,763,277.27
2011	5,227	17,394,863.78

Source: Hospital Information System of the Brazilian Health Ministry

(1US$ = R$1.81)

**Fig 2:**
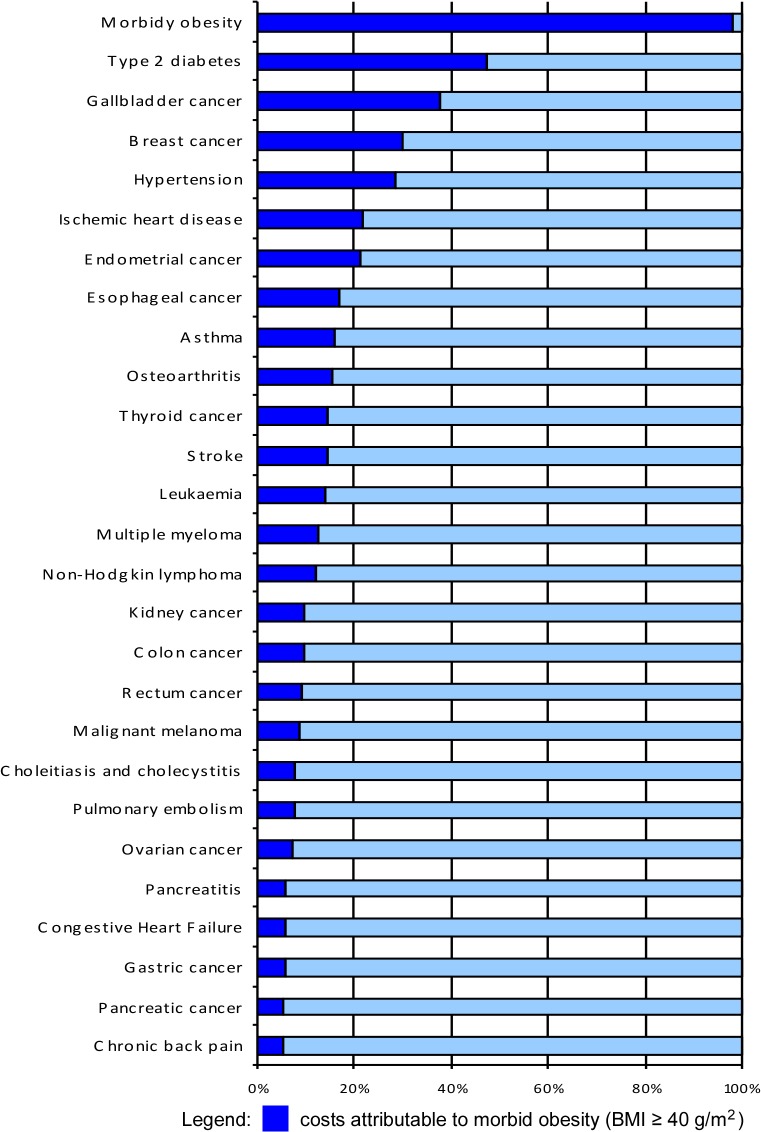
Proportion of costs attributable to morbid obesity (BMI ≥ 40 kg/m^2^) in relation to the total costs of obesity (BMI ≥ 30 kg/m^2^) for each pathology analyzed, Brazil, 2011.

Fig 2 reports the proportion of costs attributable to morbid obesity in relation to total obesity costs by comorbidity. Diabetes, gallbladder cancer, breast cancer and hypertension were found to be the diseases in which morbid obesity accounted for more than 25% of total obesity costs (Fig 2).

### Costs according to gender

In Brazil, the costs attributable to obesity in women were double the costs for men in 2011 (Table 4). Of this difference, US$ 28.8 million corresponds to female cancers (endometrial, breast, and ovarian). The cost of morbid obesity in women was five times higher than it was in men.

**Table 4:** Costs attributable to obesity and morbid obesity in the adult population aged 20 and over, according to sex and associated diseases. Brazil, 2011.

Diseases associated	Costs attributable to obesity (BMI ≥ 30 kg/m^2^) (in thousand US$)						Costs attributable to morbid obesity (BMI ≥ 40 kg/m^2^) (in thousand US$)					
	Men			Women			Men			Women		
	US$	95% CI		US$	95% CI		US$	95% CI		US$	95% CI	
Asthma	1,107	373	1,951	2,644	1,302	4,139	163	131	201	436	351	536
Breast cancer	-	-	-	16,934	6,616	28,197	-	-	-	5,085	3,220	7,207
Cholelithiasis and cholecystitis	970	94	2,036	11,764	1,799	24,270	36	3	80	956	125	2,522
Chronic back pain	3,551	3,020	4,141	7,283	6,272	8,377	108	76	148	457	323	622
Colon cancer	10,027	4,872	15,932	4,075	2,252	5,913	732	576	903	615	331	943
Congestive Heart Failure	7,345	2,377	13,546	8,935	897	18,936	283	86	567	676	61	1,660
Endometrial cancer	-	-	-	9,000	8,050	9,964	-	-	-	1,909	1,459	2,428
Esophageal cancer	5,331	3,322	7,659	2,017	1,225	2,895	710	351	1,314	519	245	966
Gallbladder cancer	105	3	233	497	353	651	3	0	9	222	3	1,122
Gastric cancer	1,494	1,025	2,531	803	550	1,359	54	37	94	76	52	132
Hypertension	1,057	666	1,493	2,794	1,310	4,490	257	224	294	835	729	949
Ischemic heart disease	30,046	21,789	38,959	61,759	55,227	68,644	3,943	-	-	16,034	-	-
Kidney cancer	897	684	1,120	1,752	1,536	1,963	57	32	93	199	130	282
Leukemia	4,091	983	7,099	9,164	2,069	8,448	311	69	595	1,528	301	3,536
Malignant melanoma	508	12,004	23,501	-	-	-	44	11	93	-	-	-
Multiple myeloma	776	342	1,294	1,017	629	1,396	64	27	114	160	96	231
Non-Hodgkin lymphoma	1,262	630	5,178	1,551	4,006	3,243	97	49	154	237	0	524
Obesity (bariatric surgery)	2,561	-	-	15,149	-	-	2,261	-	-	15,134	-	-
Osteoarthritis	3,893	2,457	5,498	3,481	3,230	3,729	202	172	234	933	801	1,080
Ovarian cancer	-	-	-	2,878	2,082	3,655	-	-	-	202	145	260
Pancreatic cancer	2,479	1,340	3,835	1,709	518	3,128	101	51	171	126	36	251
Pancreatitis	1,359	488	2,465	895	322	1,624	50	15	110	83	27	185
Pulmonary embolism	436	306	581	844	607	1,097	20	13	29	79	51	115
Rectum cancer	1,606	1,023	2,097	407	0	1,024	125	79	173	57	0	148
Stroke	2,629	1,738	3,625	3,048	1,738	4,430	245	-	-	573	-	-
Thyroid cancer	219	25	477	493	203	809	23	2	78	79	30	148
Type 2 diabetes	5,028	4,362	5,695	9,932	8,683	11,023	706	-	-	6,368	-	-
Total	88,776	63,924	150,946	180,825	111,476	223,405	10,594	-	-	53,579	-	-

(1US$ = R$1.81)

## Discussion

Costs attributable to obesity accounted for 1.86% of medium- and high-complexity healthcare expenses ($22.6 billion) in 2011[35]. Although this figure does not take into account the total cost of healthcare, it is similar to other studies. A compilation of studies of direct costs worldwide estimated that countries spend between 0.7% and 2.8% of their total health budget on obesity [36]. World Health Organization studies in Europe indicate that direct costs due to obesity generally total 2–4% of national healthcare expenses [37]. The USA is associated to considerably higher shares of the total medical healthcare costs, ranging from 5.5% to 20.1% [38].

Recently Bahia et al (2012) [39] reported on the cost of overweight- and obesity-related diseases in Brazil; however, their study was based on self-reported weights and heights from a non-representative sample (adults living in state capitals). Our analysis was based on surveys that are representative of the Brazilian population, conducted through home visits carried out by the Brazilian Institute of Geography and Statistics (IBGE) and involving more accurate height and weight measurements. If we compare the obesity-attributable costs obtained, we see that our study calculated higher costs, which is probably due to its more accurate estimation of obesity prevalence and the higher number of comorbidities analyzed. Another obesity study conducted by Sichieri et al. [40] estimated the cost to the Brazilian public health system of inpatient care for individuals aged 20 to 60 associated with overweight and obesity and their comorbidities in 2001. The costs totaled $36 million, representing 3.02% of total inpatient costs for men and 5.83% for women. The amounts found in our study were five times higher ($157.6 million) due to the increased prevalence of obesity in this period and the inclusion of the elderly (over 60 years of age).

When comparing the obesity costs of different countries, it is important to consider not only the differences in methodologies used and in population and epidemiological characteristics, but also the fact that the healthcare systems and services provided vary greatly from country to country and even between regions of the same country [26]. It is also important to emphasize that age and sex influence the results of studies into the relationship between obesity and healthcare costs [36].

Turning to comparative costs between the sexes, our finding that the cost of obesity is higher in women has also been identified in other studies of the cost of obesity in Brazil [39, 40]. However, in our analysis the cost of obesity in women was found to be proportionally higher. This result can be explained by several factors: a) a higher prevalence of obesity and especially morbid obesity in women; b) women use health services more than men [39]; c) US$ 28.8 million was attributable to three exclusively female types of cancer (breast, ovarian and endometrial); and d) the cost of morbid obesity in women was five times higher than in men, probably because most of the relative risks (or OR) were higher in women with morbid obesity and most bariatric surgeries conducted by the public health system in 2011 were for women.

The costs related to bariatric surgery totaled $17.4 million (27.1% of morbid obesity costs). Compared with the findings of a study [41] conducted between 1999 and 2006, we found that in 2011 more bariatric surgeries were done than in previous years, resulting in higher costs for the Brazilian health system.

It is generally accepted that the Brazilian public health system has a large unmet demand for bariatric surgery. It is estimated that 1.5 million adults in the country have morbid obesity and only 26,853 surgeries were performed between 2003 and 2011 in the Brazilian public health system [5, 35]; this in a country where about 75% of the population are dependent on public health services.

When we compare the cost of the disease with its prevalence in the population, we find that the cost of morbid obesity (with a prevalence of 0.81% and a cost of $64.2 million) was proportionally 4.3 times higher than the cost of obesity (with a prevalence of 14.8% and a cost of $269.6 million). This is consistent with the findings of other studies[1, 27, 42, 43], which show a direct correlation between rising costs and higher BMI, except that we found the proportion of costs related to morbid obesity to be even higher than in other surveys [44–46]. In this study, it was not possible to calculate per capita costs as the Brazilian health information systems use procedures, not individuals, as a unit of analysis, since they were developed for the purposes of reimbursing costs of procedures rather than for epidemiological analyses.

According to Andreyeva et al.[45], the healthcare cost of individuals with a BMI of over 40 kg/m² is twice as high as it is for normal weight people. A study in the United States found that the annual per capita cost for morbidly obese adults was 81% ($1,975) higher than for eutrophic adults (CI: 95%, 48–121%), 65% ($1,735) higher than for overweight individuals (CI: 95%, 37–110%), 47% ($1,415) higher (CI: 95%, 11–96%) than for obese adults (BMI≥ 30 kg/m^2^) and 25% ($888) higher (CI: 95%, 2.3–52%) than for Class II obese adults (BMI ≥ 30 kg/m^2^ and ≤ 34.9 kg/m^2^) [27]. Another study [47] found the cost of obesity compared to normal weight individuals (BMI 20–24.9 kg/m^2^) to be 21–54% higher for BMI 30–35 kg/m^2^; 43–57% higher for BMI 35–40 kg/m^2^; and 78–111% higher for BMI ≥ 40 kg/m^2^. A systematic review of direct obesity-related healthcare costs conducted by Tsai et al [48] identified five studies that reported cost estimates for morbid obesity (BMI ≥ 40 kg/m^2^). The average cost increase was $3,012, which was 68% higher than for people of normal weight and 35% higher than for all obese people (variation between 25% and 49%).

This analysis did not aim to calculate the indirect costs of obesity (sick leave, reduced productivity at work, premature death), but it is important to remember that there are other costs involved with this health problem. Obese people tend to be hired less, earn lower wages and take longer to be promoted than people of normal weight, as well as less likely to receive appropriate preventative healthcare such as cancer screening tests [22]. Obese individuals can suffer from reduced quality of life, depression, anxiety and low self-esteem, characteristics that are more common among extremely obese people [22]. These are just some examples of the indirect and intangible costs attributable to obesity that also need to be considered in a more comprehensive analysis. For example, when compared to normal weight individuals (20 kg/m^2^ ≤ BMI < 25 kg/m^2^), obese adults (BMI ≥ 30 kg/m^2^) have been found to make 38% more visits to primary care doctors and moderately and severely obese people have been found to have 34% and 74% more days of hospitalization, respectively [49]. Also, individuals with a BMI of greater than 30 kg/m^2^ have been found to receive 1.84 more medication dispensations in general and 3.4 times more for cardiovascular diseases [49].

When obesity prevention strategies fail (physical activity, intake of healthier foods, multidisciplinary care, medication), bariatric surgery can still be an effective way of reducing morbi-mortality and costs. Crémieux et al [50] estimated the return on investment for bariatric surgery from the insurer’s perspective in the United States, indicating that costs were fully recovered after 53 months; when the procedure was laparoscopic rather than laparotomic, the cost recovery period dropped to 25 months. In Brazil, Sussenbach et al [51] showed that bariatric surgery reduces associated diseases: 36 months after surgery, patients showed 97% resolution for type 2 diabetes mellitus, 98% for systemic arterial hypertension and 95% for dyslipidemias. In terms of costs, the authors found that average costs (medication, professional care, examination) after surgery were substantially lower than before it: 36 months after bariatric surgery, costs reduced to 30% for patients without comorbity, 19% for patients with one comorbity, and 32% for patients with two or more comorbities [51].

### Study limitations

We understand that it would be ideal to use relative risks (RR) from studies conducted in Brazil. However, while the absolute prevalence of some obesity-related diseases varies from country to country, the relative risk of any particular disease (risk for obese people compared with risk for normal weight people) is quite similar around the world [52]. Despite the fact that the PAR for morbid obesity is probably greater than for obesity for diseases such as ovarian cancer, gastric cancer, congestive heart failure, pulmonary embolism, pancreatitis, chronic back pain, pancreatic cancer, gallbladder cancer, cholecystitis and cholelitiasis, the same PAR value for obesity was adopted to calculate morbid obesity costs since we could not find the RR associated with these pathologies in the BMI ≥ 40 kg/m^2^ range. It is also important to consider that not all the expenses incurred by the public health system are recorded in information systems, such as funds transferred for the distribution of medicines for diabetes and hypertension in the primary healthcare network.

## Conclusion

The direct cost of obesity in adults to the public health system in Brazil in 2011 was estimated at US$ 269.6 million. This covers the cost of medium- and high-complexity procedures involved in treating obesity and diseases attributable to obesity. These costs attributable to obesity corresponded to 1.86% of all Ministry of Health’s expenditure related to hospital and ambulatory care in Brazil (59.2% of the total was hospital admissions and 40.8% corresponded to ambulatory procedures). The costs attributable to obesity in women were double the costs for men and the cost of morbid obesity in women was five times higher than it was in men. The obesity-attributable costs were highest for ischemic heart disease, followed by breast cancer, congestive heart failure and diabetes.

When analyzed by severity, the cost of morbid obesity was found to be proportionally higher than the cost of obesity. Despite its low prevalence in the population (0.81%), morbid obesity now accounts for almost a quarter of the costs attributable to obesity in the public health system, totaled $64.2 million in 2011. Bariatric surgery was the single highest cost, accounting for $17.4 million, or 27.1% of costs attributable to morbid obesity.

If the current epidemic were not reversed, the prevalence of obesity in Brazil will increase gradually in the coming years, as well as its costs, having serious implications for the financial sustainability of the public health system. With regard to public policies, it is essential to strengthen the actions already started in Brazil for the prevention and control of obesity, such as: a) increasing the supply and availability of healthy foods, including the reduction of prices of fruit and vegetables; b) the promotion of healthy environments to facilitate adequate food choices and physical activity; c) adoption of effective interventions at all points of the health care network in order to ensure the completeness of obesity care in the public health system; d) food and nutrition education, as the publication of the Food Guide for the Brazilian population and its dissemination; e) restriction of unhealthy food marketing, especially for children.

Studies of this nature need to be conducted on a regular basis in Brazil in order to monitor the economic impacts of the obesity epidemic in the coming years.
